# Microwave‐Assisted Synthesis of SrTiO_3_ Nanocuboids without TiCl_4_

**DOI:** 10.1002/smsc.202200107

**Published:** 2023-04-06

**Authors:** Ian L. Peczak, Robert M. Kennedy, Aili M. Simpson, Massimiliano Delferro, Kenneth R. Poeppelmeier

**Affiliations:** ^1^ Department of Chemistry Northwestern University Evanston IL 60208 USA; ^2^ Chemical Sciences and Engineering Division Argonne National Laboratory 9700 S. Cass Avenue Lemont IL 60439 USA

**Keywords:** catalyst supports, heterogeneous catalysis, hydrothermal, microwave-assisted, nanoparticles, plastic upcycling, strontium titanate

## Abstract

Strontium titanate (STO) nanocuboids are a novel support for Pt nanoparticle catalysts (Pt/STO). The first of many steps in commercializing Pt/STO will be developing a scalable, environmentally sustainable, and cost‐effective STO nanocuboid synthesis. Herein, Sr–Ti–OH mixtures are synthesized from various Sr^2+^ and Ti^4+^ reagents and treated by convection‐ and microwave‐assisted heating to obtain STO nanoparticles. These experiments clarify how phase composition of the prehydrothermal Sr–Ti–OH mixture and choice of heating method affect final nanoparticle morphology. In Sr–Ti–OH mixtures synthesized with TiCl_4_, STO is the most stable phase and precipitates prior to heating, while titania sol–gels are the most stable phase when other Ti^4+^ sources are used. STO nanocrystals always form when Sr–Ti–OH mixtures are treated by convection heating, though nanocuboids are only observed if STO precipitates from the Sr–Ti–OH mixture. Microwave‐assisted hydrothermal treatment more rapidly heats the precursor solution, and thus, STO nanocuboids can form from a variety of Sr–Ti–OH mixtures regardless of mixture composition. Two microwave syntheses of STO nanocuboids are reported: one which uses TiCl_4_ as a Ti^4+^ source, and another that uses titanium(IV) bis(ammonium lactato) dihydroxide ([NH_4_CH_3_CH(O)CO_2_]_2_Ti(OH)_2_), a water‐stable Ti^4+^ complex.

## Graphical abstract

The synthesis of strontium titanate (STO) catalyst supports is studied as part of a larger effort to synthesize waste plastic upcycling catalysts in a scalable fashion. A microwave‐assisted heating method is developed to afford 20 + g STO samples using TiBALD, a water‐stable Ti^4+^ source whose use facilitates reaction scale‐up.

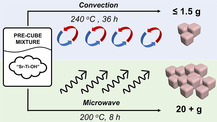

## 1 Introduction

Modern chemical industry is highly dependent on catalysts, with most chemical processes requiring their use in one or multiple steps.^[^ ^1^ ^]^ By some accounts, over a third of the world's gross domestic product (GDP) is tied to materials generated by catalysts, ≈85% of which are heterogeneous.^[^ ^1^, ^2^ ^]^

Owing to the proven success of catalysts in industrial processes, many researchers are using catalysts at the lab scale to solve unexplored problems.^[^ ^3^, ^4^, ^5^ ^]^ One such problem is the chemical recycling of single‐use plastics, as plastic pollution is a pressing global crisis that urgently needs an industrial solution.^[^ ^6^, ^7^, ^8^, ^9^ ^]^ Processing single‐use plastic waste is uniquely challenging because the stable carbon–carbon and carbon–hydrogen bonds that make up these materials are difficult to break.^[^ ^10^ ^]^ However, catalytic upcycling processes can potentially deconstruct plastics into uniform, value‐added products, thereby converting waste into economically viable materials that close the loop of a circular economy.^[^ ^11^, ^12^ ^]^ Thus far, one state‐of‐the‐art technique for waste plastic upcycling is catalytic hydrogenolysis, by which a noble metal nanoparticle catalyst (e.g., Pt, Ru) deposited on a metal oxide support (e.g., perovskites, titania, silica) selectively converts waste polyolefin samples to liquid and wax‐like products.^[^ ^13^, ^14^, ^15^, ^16^, ^17^, ^18^, ^19^, ^20^ ^]^ Among such catalysts, platinum nanoparticles (Pt) on SrTiO_3_ nanocuboid supports (STO; Pt/STO) have stood out for their demonstrated ability to selectively convert virgin and postconsumer polyethylene and polypropylene into commercially viable base oils for tribological applications.^[^ ^13^, ^19^, ^20^ ^]^ Thus, continued study of Pt/STO is desirable.^[^ ^21^, ^22^, ^23^, ^24^, ^25^ ^]^

The development of a scalable and cost‐effective synthesis for STO catalyst supports is one of many milestones that must be met if Pt/STO hydrogenolysis catalysts are to upcycle plastic waste on a scale sufficient for participation in a circular economy. Regarding STO supports, current lab‐scale syntheses use reagents not suitable for eventual scale‐up.^[^ ^26^ ^]^ Of the literature reports that demonstrate hydrothermal synthesis for highly cubic STO samples (defined here and in our previous work as a batch of nanoparticles with >80% nanocuboids),^[^ ^24^ ^]^ the majority use titanium tetrachloride (TiCl_4_) as a source for Ti^4+^.^[^ ^26^, ^27^, ^28^, ^29^, ^30^, ^31^, ^32^ ^]^ Chloride ions, as well as other halides, have a demonstrated track record of acting as both inhibitors and promoters in a variety of catalytic processes. Specifically, chloride ions can be poisons in the oxidation of CO, methane, ethane, and crotonaldehyde hydrogenation, and generally appear to deactivate platinum catalysts.^[^ ^33^, ^34^, ^35^ ^]^ Interestingly, they also promote selective ethene epoxidation and cyclohexene hydrogenation, among other examples.^[^ ^36^, ^37^ ^]^ Compounding these issues, chloride ions have been shown to corrode steel‐based reactors and significantly shorten equipment lifetime.^[^ ^38^, ^39^, ^40^ ^]^ Thus, TiCl_4_ restricts the application of STO and other materials as catalyst supports for polymer upcycling. Finally, the introduction of TiCl_4_ into an aqueous reaction mixture requires the use of ethanol to stabilize the compound. Titanium (IV) chloride is reactive in air, and reagent stocks often decompose to titanium‐containing oxides over time, again restricting its ability to serve as a precursor for STO synthesis. This increases the frequency with which reagents must be replaced and drives up the materials cost required to make STO. Preliminary estimations also suggest that for a hypothetical STO synthetic process, a significant amount of greenhouse gas emissions stem from the use of ethanol.^[^ ^41^ ^]^ Thus, it is highly desirable to substitute TiCl_4_ for an alternate Ti^4+^ source to avoid potential corrosion, increase precursor shelf life, and eliminate the use of ethanol to reduce the carbon footprint of the process.

Choosing an effective heating method for hydrothermal treatment may also impact the viability of a large‐scale STO synthesis. Currently, many STO syntheses for nanocuboids use convection heating in hydrothermal treatment steps.^[^ ^26^, ^27^, ^28^, ^29^, ^30^, ^31^, ^32^, ^42^, ^43^, ^44^ ^]^ Convection heating (CH) relies on the diffusion of thermal energy through a reactor and into the reaction mixture, which may cause inhomogeneous sample heating. This can extend the heating time required to synthesize a highly cubic STO sample, consuming large amounts of energy and potentially allowing for more particles with irregular morphologies to form.

Microwave‐assisted hydrothermal (MAH) heating more uniformly distributes energy throughout reaction mixtures compared to convection‐based heating.^[^ ^45^, ^46^, ^47^, ^48^, ^49^, ^50^, ^51^, ^52^, ^53^, ^54^, ^55^, ^56^ ^]^ For solvents that are microwave active, e.g., water, direct delivery of microwave radiation into a reaction mixture via a magnetron‐powered thermocouple causes molecular rotation. This produces uniform heating throughout the reaction medium, thereby cutting down on reaction times and making batch processes more amenable to scale‐up.^[^ ^45^, ^46^, ^47^, ^48^, ^49^, ^50^, ^51^ ^]^ Accordingly, microwave reactors have been used extensively in organic synthesis to synthesize small‐molecule samples in as little as 30 min.^[^ ^46^ ^]^ They have significantly shortened the time required to conduct transition‐metal‐catalyzed carbon–carbon and carbon–heteroatom bond‐forming reactions, such as Heck, Stille, Negishi, and Suzuki couplings with palladium catalysts, Ullman condensations, and carbonylations, among other processes.^[^ ^51^ ^]^ Moreover, these small molecule heating techniques have been adapted to accelerate syntheses in adjacent fields, such as in high‐speed polymerase chain reactions and enzyme‐mediated organic transformations.^[^ ^46^, ^51^ ^]^ However, they have not been widely adopted because a limited number of solvents are microwave active, and the cost of microwave reactors is significantly higher than that of convection heating equipment.

Microwave heating is also used to produce inorganic materials, though these syntheses are less common than their organic analogs because they often require harsh reaction conditions.^[^ ^45^, ^47^ ^]^ Generally, microwave heating has been used to synthesize most classes of functional materials, including but not limited to oxides, halides, and sulfides.^[^ ^52^, ^53^, ^54^, ^55^, ^56^ ^]^ These studies have largely focused on demonstrating proof‐of‐concept syntheses, which do not routinely select for certain physical properties of the product or demonstrate output beyond the lab scale. Thus, there is a continued need to investigate how MAH techniques can be used both for scale‐up and the synthesis of specific nanoparticle morphologies.

In this work, a variety of Ti^4+^ and Sr^2+^ precursors were used to synthesize prehydrothermal Sr–Ti–OH mixtures, which were analyzed by X‐ray diffraction to determine how Sr–Ti–OH phase composition impacts final STO particle morphology. The results are consistent with a previous report by Peczak et al. that suggests STO nanocuboids form in a two‐step process that requires: 1) precipitation of STO and 2) {100} facet growth.^[^ ^24^ ^]^ When TiCl_4_ is not used as a Ti^4+^ precursor, nanocuboid particles generally do not form when a single‐step convection heating profile is used. Additionally, STO nanocuboids were synthesized in a 1 L stirred microwave reactor to explore both reaction scale‐up and the impact of heating method on final particle morphology. These syntheses were first conducted using TiCl_4_ as a Ti^4+^ source, and then conducted by replacing TiCl_4_ with titanium(IV) bis(ammonium lactato)dihydroxide (TiBALD), a water‐stable titanium complex. From these reactions, 20 g of highly cubic STO were obtained in less time and at lower temperatures than previously required (200 °C, 8–16 h). Thus, we demonstrate that to synthesize STO nanocuboid samples via convection heating, a Ti^4+^ precursor must be chosen such that STO crystallites precipitate prior to heating. However, through the newly developed microwave‐assisted synthesis reported herein, STO nanocuboids can now be synthesized from more Ti^4+^ sources than previously possible.

## 2 Results and Discussion

### 2.1 Phase Composition of Prehydrothermal Sr–Ti–OH Mixtures

Previously, Peczak et al. demonstrated that to obtain a highly cubic STO sample after a one‐step convection heat treatment, STO nanocrystals must precipitate prior to hydrothermal synthesis.^[^ ^24^ ^]^ For the reported synthesis, which used Sr(OH)_2_.8H_2_O and TiCl_4_ as starting reagents, this was accomplished by specifying an order of operations to ensure that the prehydrothermal Sr–Ti–OH reaction mixture (“Sr–Ti–OH”) rapidly reached pH 14. Under these conditions, STO is the thermodynamically most stable phase and therefore precipitates. Subsequent hydrothermal heating is likely responsible for {100} facet growth on particles, which affords the final highly cubic product. To explore whether this phenomenon occurs in other reaction systems, Sr–Ti‐OH reaction mixtures made with varied starting sources of Sr^2+^ and Ti^4+^ were synthesized without heating and then isolated and analyzed by powder X‐ray diffraction. All Sr–Ti–OH mixtures were created using 10 M NaOH.

Figure S1, Supporting Information, presents a graphical depiction of a “stoplight” screening method used to compare new Sr–Ti–OH mixtures (synthesized from seven Ti^4+^ sources and seven Sr^2+^ sources with Sr(OH)_2_.8H_2_O and TiCl_4_ serving as a control) to that previously reported by Peczak et al. By this method, Sr–Ti–OH reaction mixtures from which only crystalline STO precipitated (>99.9%, as determined by powder X‐ray diffraction) were considered satisfactory (i.e., green, Figure S2a, Supporting Information), as these mixtures can be converted into highly cubic products with a one‐step convection heat treatment. Sr–Ti–OH reaction mixtures from which either amorphous or weakly crystalline materials precipitated (as determined by the final X‐ray diffraction pattern) were considered less desirable (i.e., red, Figure S2b, Supporting Information).

First, Sr(OAc)_2_, Sr(Oac)_2_.0.5H_2_O, Sr(NO_3_)_2_ and SrCO_3_, Sr(ClO_4_)_2_.3H_2_O, and SrCl_2_.6H_2_O were used to synthesize a Sr–Ti–OH reaction mixture, which was then analyzed to determine phase composition. Molar amounts of each Sr^2+^ starting reagent were chosen to meet a global ratio of 1.05:1 of Sr to Ti, and the average crystallite size for the primary phase of each mixture was measured by a Debye Scherrer peak broadening analysis. TiCl_4_ was used as the Ti^4+^ source for all samples in these experiments. The findings are displayed in **Table** **1**. Several Sr–Ti–OH mixtures derived from various Sr^2+^ sources precipitated STO without any secondary phases. When Sr(OAc)_2_, Sr(OAc)_2_.0.5H_2_O, and SrCO_3_ were used as the Sr^2+^ source, STO crystallites precipitated from solution, while the NO_3_^−^, ClO_4_^−^, and Cl^−^ anions prevented STO precipitate formation. For all samples that precipitated STO, average STO crystallite sizes were between 18 and 24 nm, which is generally equivalent to those measured from Sr–Ti–OH mixtures synthesized with Sr(OH)_2_.8H_2_O and TiCl_4_, as previously reported (repeated in Table 1).

**Table 1:** Variation of Sr^2+^ source and resulting crystalline composition of final Sr–Ti–OH mixture. All Sr–Ti–OH mixtures were synthesized with the designated Sr^2+^ reagent in 1 M CH_3_COOH in water, with TiCl_4_ as a Ti^4+^ source in 20 mL ethanol, and 15 mL of 10 M NaOH as a source of base. Average crystallite size was measured in MDI Jade by peak broadening analysis^[^ ^89^ ^]^

Sr^2+^ source	% STO	% SrCO_3_	Average crystallite size [nm]
Sr(OH)_2_.8H_2_O	>99.9	0	20.7 ± 4.6
Sr(NO_3_)_2_	18.9	81.1	23.5 ± 7.0
Sr(OAc)_2_	>99.9	0	24.0 ± 9.1
Sr(OAc)_2_.0.5H_2_O	>99.9	0	18.3 ± 6.7
Sr(ClO_4_)_2_.3H_2_O	12.0	88.0	28.3 ± 6.7
SrCO_3_	>99.9	0	24.4 ± 7.3
SrCl_2_.6H_2_O	0	100	24.8 ± 5.4

Literature reports propose a variety of mechanisms by which STO nanoparticles might form,^[^ ^57^ ^]^ and two of the most common pathways proposed are the dissolution–precipitation and in situ transformation mechanisms.^[^ ^26^, ^57^ ^]^ Though there is ample evidence for each process, both require Sr^2+^ and Ti^4+^ to combine in solution. Previous reports also note that Sr^2+^ may incorporate into hydrolyzed, Ti‐containing structures as a prerequisite to precipitation.^[^ ^43^ ^]^

The precipitation of 100% crystalline STO from four Sr–Ti–OH reaction mixtures in Table 1 suggests that in these four mixtures, STO is the most thermodynamically stable phase, as has been observed previously in analogous systems.^[^ ^24^ ^]^ In the case of the three Sr–Ti–OH reaction mixtures with a composition of 100% SrCO_3_, the precipitation of this strontium carbonate crystalline phase could be the result of a decrease in Sr–Ti–OH reaction mixture pH. Sr–Ti–OH reaction mixtures are highly basic, and so a non‐negligible amount of CO_2_ can be uptaken from air and lower solution pH to an intermediate range, making SrCO_3_ the thermodynamically preferred phase instead of STO. Additionally, it is possible that SrCO_3_ precipitation occurs because anions (e.g., NO_3_^−^, ClO_4_^−^) sterically interfere with the interaction of Sr^2+^ cations and Ti^4+^ or the hydrolysis of Ti^4+^. Such cases can allow sufficient time for the local formation of SrCO_3_ crystallites and amorphous titania. These phases would then precipitate and leave no metal ions from which to form STO.^[^ ^43^ ^]^ A more thorough investigation of this phenomenon would require time‐resolved, pH‐controlled studies that track the evolution of various strontium and titanium species.

For the mixtures in Table 1, it is possible that for Sr–Ti–OH samples in which STO does not precipitate, a change in solution pH during mixing, which would no longer make STO the dominant phase and drive the formation of other compounds, or steric interference, which would prevent the interaction of Sr^2+^ and Ti^4+^ ions, occurs. For samples that did not precipitate STO, the counterions for each strontium reagent are the conjugate base of a strong acid, meaning that their presence likely does not alter the solution pH during mixing. Thus, NO_3_^−^, ClO_4_^−^, and excess Cl^−^ may sterically interfere with the interaction of Sr^2+^ and Ti^4+^ in solution. A more thorough investigation of this phenomenon requires time‐resolved, pH‐controlled studies that track the evolution of various strontium and titanium species, as has been done previously for titania sol–gel systems.

**Figure 1:**
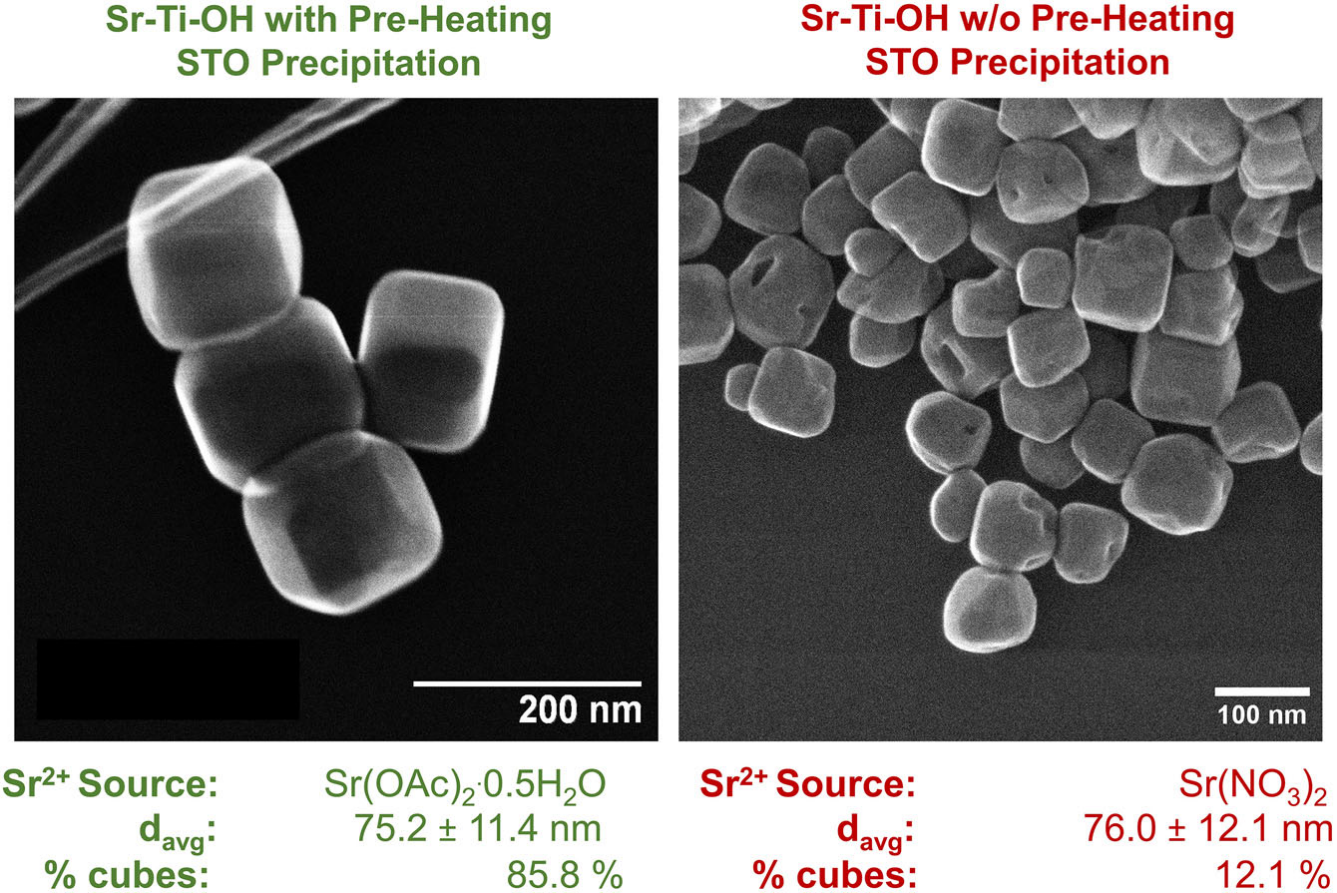
Secondary‐electron STEM images of STO samples after hydrothermal treatment of Sr–Ti–OH mixtures that did (left) and did not (right) precipitate STO prior to heating. The sample that precipitated STO prior to heating was synthesized using Sr(OAc)_2_ as a Sr^2+^ source, while the sample that did not precipitate STO prior to heating was made using Sr(NO_3_)_2_ as a Sr^2+^ source. Both samples were made using TiCl_4_ as a Ti^4+^ source. The results suggest that for unstirred convection heating in one‐step heating profiles, STO nucleation prior to hydrothermal treatment is critical for the formation of highly cubic samples. For each sample above, 300 nanoparticles were imaged and analyzed to calculate the percentage of nanocuboids per sample, while 200 nanoparticles were imaged and analyzed to determine average particle size.

Two of the Sr–Ti–OH mixtures in Table 1 were treated hydrothermally for 36 h at 240 °C in a 125 mL autoclave in a convection oven. The initial Sr–Ti–OH mixtures were characterized by X‐ray diffraction, and the final products were characterized by both X‐ray diffraction and electron microscopy (**Figure** **1**, Figure S2, Supporting Information). While both samples had crystalline compositions of >99.9% STO, only the sample that precipitated STO prior to hydrothermal treatment produced a highly cubic final sample. These results support the previous conclusion that STO nanocuboids form in a two‐step precipitation and facet growth process and suggest that it is independent of reagent identity. The formation of the nanocuboid shape, which changes both the chemical (e.g., surface hydroxyl concentration) and physical (e.g., faceting) properties of the nanoparticle surface, is critical for use of STO as a catalyst support because it allows for epitaxial stabilization of Pt on STO, thereby preventing sintering during catalysis. Given that a beveled nanocuboid is the Wulff shape of STO in water, a one‐step convection heating profile is likely not sufficient to drive both STO precipitation and facet growth because all STO nanoparticles should rearrange to the Wulff shape under sufficient temperature and time.

**Figure 2:**
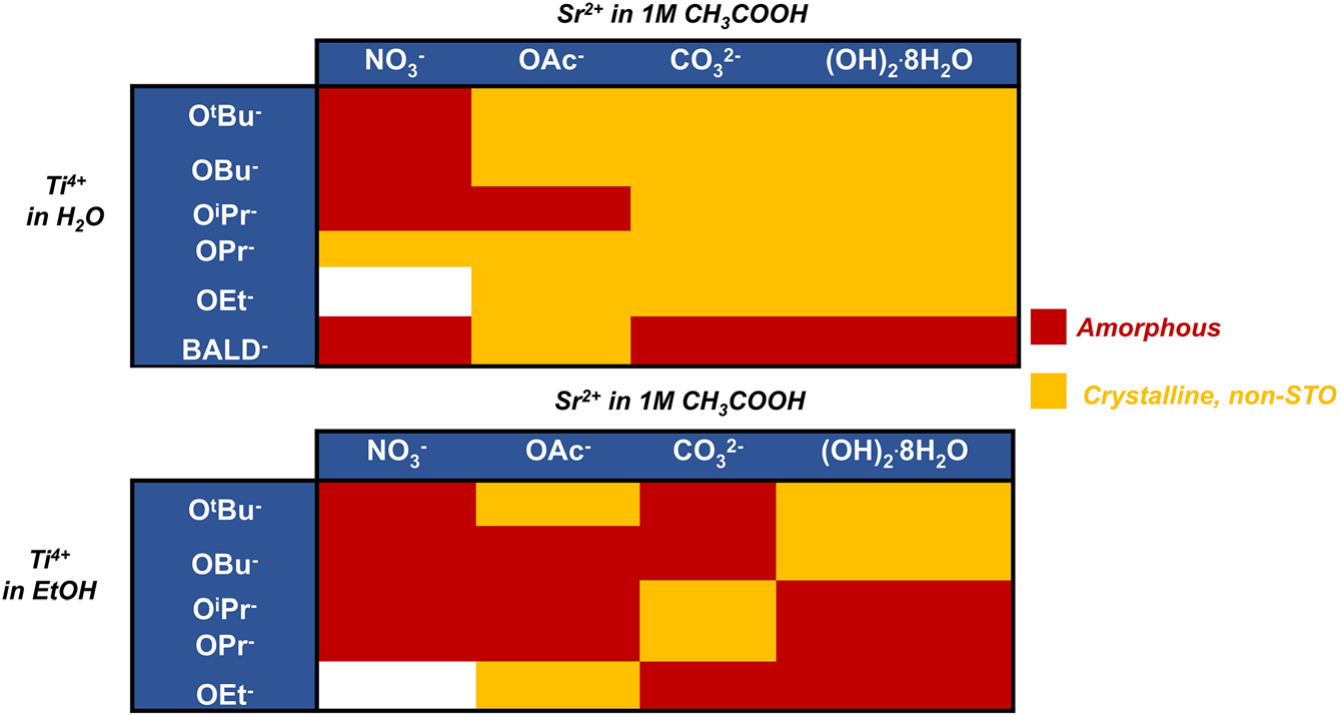
Powder X‐ray diffraction characterization of Sr–Ti–OH reaction mixture precipitates (varied Sr^2+^ and Ti^4+^ sources, with and without ethanol as a solvent). Six Ti^4+^ sources were tested for comparison against TiCl_4_, which serves as a control against which to benchmark the results above.^[^ ^24^ ^]^ None of the combinations tested precipitated STO prior to hydrothermal treatment. All samples were either amorphous, weakly crystalline, or crystallized a product other than STO. Boxes that are colored white denote a combination that was not tested. Phase determination was conducted using MDI Jade software.^[^ ^89^ ^]^

Next, analogous screening experiments were conducted by synthesizing Sr–Ti–OH mixtures with various Ti^4+^ sources. Four strontium reagents (Sr(OAc)_2_, SrCO_3_, Sr(NO_3_)_2_, and Sr(OH)_2_.8H_2_O) were each used to synthesize gel mixtures with sodium hydroxide and one of five titanium sources: titanium ethoxide (Ti(OEt)_4_), titanium propoxide (Ti(OPr)_4_), titanium butoxide (Ti(OBu)_4_), titanium *tert*‐butoxide (Ti(O*t*Bu)_4_), and titanium(IV) bis(ammonium lactato) dihydroxide (TiBALD). Titanium alkoxide sources were used because they did not require harsh conditions for dissolution in water or ethanol. The strontium sources chosen were those that successfully precipitated STO prior to heating in previous experiments. Sr(NO_3_)_2_ was also tested to determine whether samples that did not precipitate STO when mixed with TiCl_4_ could precipitate STO when mixed with different reagents. Each mixture was separated from its supernatant by centrifugation and analyzed by powder X‐ray diffraction. These Sr–Ti–OH mixtures were synthesized both with and without ethanol, and experiments are grouped as such in the top and bottom halves of **Figure** **2**, respectively. Each strontium or titanium reagent is labeled by the anion to which it corresponds, i.e., a sample synthesized with titanium *tert*‐butoxide is listed in the row labeled “O^*t*^Bu^−^,” and a sample synthesized with strontium carbonate is listed in the column labeled “CO_3_^2−^.”

STO crystallites never precipitate from Sr–Ti–OH reaction mixtures that are synthesized with titanium sources other than TiCl_4_. All permutations tested formed samples that were either amorphous, weakly crystalline, or a crystalline phase that was not STO. Of the samples that were crystalline, all observed phases were either unwanted side products, such as SrCO_3_, or crystalline byproducts that could not be identified, likely sodium–titanium complexes. Figure S2a,b, Supporting Information, shows representative diffraction patterns that are 100% STO and weakly crystalline SrCO_3_, respectively. Additionally, the following representative diffraction patterns are provided in the Supporting Information with important features noted: an amorphous diffraction pattern (Figure S3a, Supporting Information), a weakly crystalline diffraction pattern with phases other than SrCO_3_ (Figure S3b, Supporting Information), and a crystalline diffraction pattern that is likely reformed Sr(OH)_2_.8H_2_O (Figure S3c, Supporting Information).

The use of TiCl_4_ as a Ti^4+^ source appears to be crucial to precipitating STO. This is likely because generation of hydrochloric acid produces a low pH in the titanium–ethanol solution. The low pH may stabilize Ti^4+^ complexes in solution prior to mixing with NaOH and therefore prevent the creation of a solution with intermediate pH, where other phases are more stable.^[^ ^58^, ^59^, ^60^, ^61^, ^62^, ^63^ ^]^ Specifically, the titanium alkoxide compounds used in the experiments reported in Figure 2 are known to form amorphous, polymeric networks in solution, often sol–gel structures, because the metal–oxygen bond is susceptible to nucleophilic attack.^[^ ^64^, ^65^, ^66^, ^67^, ^68^, ^69^, ^70^, ^71^, ^72^, ^73^, ^74^, ^75^, ^76^ ^]^ For systems containing Ti^4+^ at low pH, such as Sr–Ti–OH mixtures made with TiCl_4_, previous reports suggest that Ti^4+^ adopts a variety of soluble hydrolyzed and semihydrolyzed forms (e.g., Ti(OH)_2_^2+^, Ti(OH)_3_^+^, and Ti(OH)_4_), which likely prevents other structures from forming.^[^ ^77^, ^78^, ^79^, ^80^ ^]^ If TiCl_4_ is used, there are no competing anions to interfere with STO precipitation. The alkoxide ligands mentioned previously are conjugate bases to weak acids, and the weak acids likely form in solution upon creation of the Sr–Ti–OH mixture through the consumption of protons, which raises the solution pH and creates an environment (3 < pH < 9) in which sol–gel structures are more stable than STO.^[^ ^24^, ^81^ ^]^ Moreover, in this pH range, SrCO_3_ is the most stable strontium‐containing phase,^[^ ^81^ ^]^ and trace amounts of it were observed in some of the weakly crystalline powder patterns corresponding to the experiments in Figure 2. Sr–Ti–OH mixtures synthesized with the TiBALD solution do not react with water because the complexing ligands stabilize Ti^4+^ against nucleophilic attack.^[^ ^44^ ^]^ However, the corresponding diffraction patterns were amorphous because the bidentate ligands coordinated to the Ti metal center likely stayed coordinated during mixing, preventing the interaction of Sr^2+^ and Ti^4+^ to form STO.

**Figure 3:**
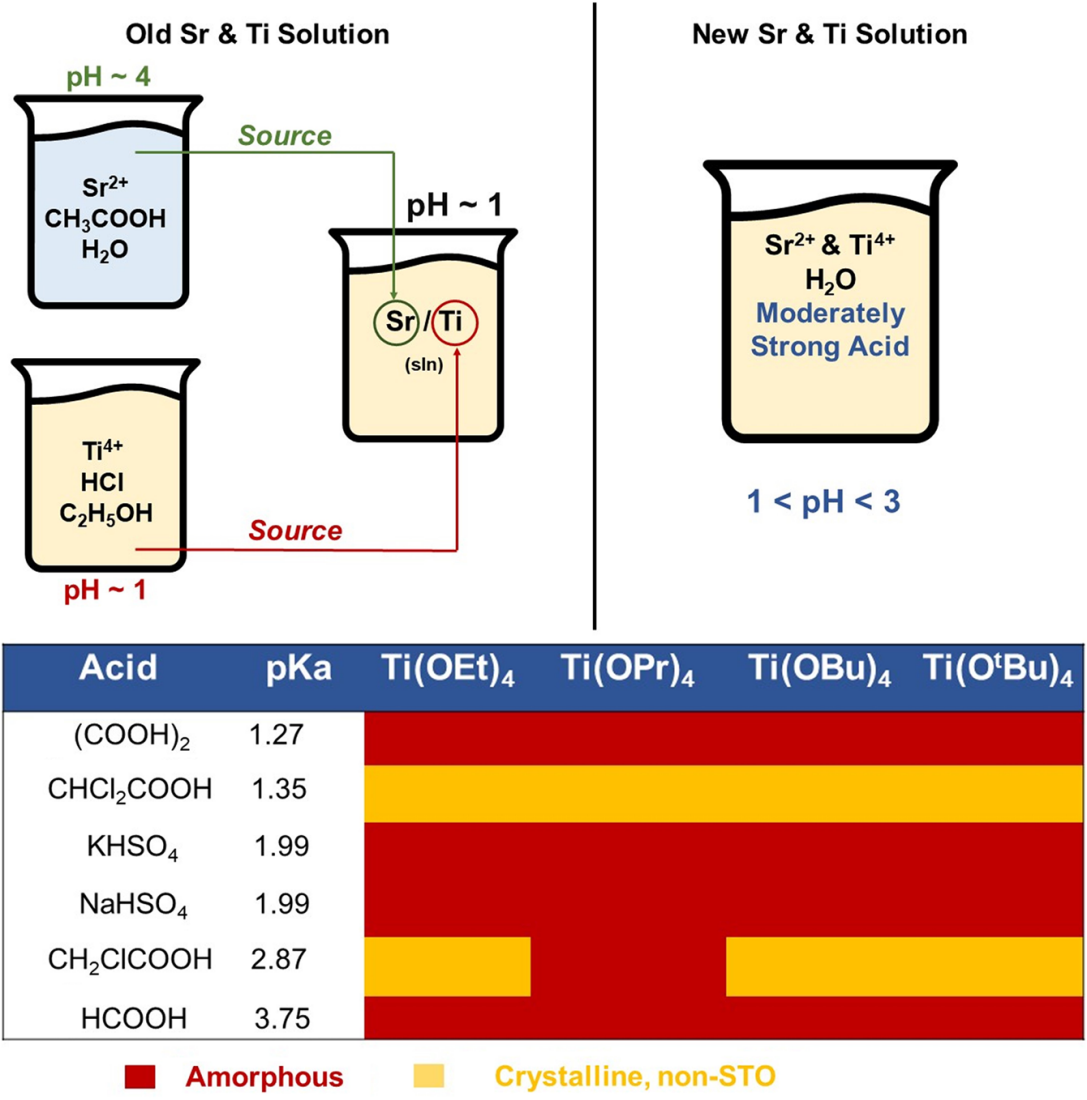
Sr–Ti–OH reaction mixtures synthesized from a bimetallic Sr/Ti solution (no ethanol, acetic acid substituted for a moderately strong acid). In place of a two‐solvent bimetallic Sr/Ti solution, in which 1 M CH_3_COOH in H_2_O dissolves an Sr^2+^ source and TiCl_4_ is dissolved in ethanol, a Sr/Ti solution was created with only water and a moderately strong acid with a p*K*_a_ value between 1 and 4. These Sr–Ti–OH reaction mixtures were synthesized by dissolving the Sr^2+^ precursor in 50 mL water and 1 m of the chosen acid in H_2_O, after which the corresponding Ti^4+^ alkoxide was added. The resulting Sr–Ti–OH mixture was isolated and analyzed by powder X‐ray diffraction. All samples were either amorphous or weakly crystalline. Phase compositions were determined using MDI Jade software.^[^ ^89^ ^]^

Based on conclusions drawn from the results in Figure 2, lowering the solution pH below 3 in a Sr/Ti solution made with titanium alkoxide complexes may drive the precipitation of STO in the final pH 14 Sr–Ti–OH mixture. Currently, an important source of acidity in the synthesis is acetic acid (p*K*_a_ = 4.76), which is used to dissolve the Sr^2+^ precursor in water. In place of acetic acid, several moderately strong acids with lower p*K*_a_ values were tested to determine whether STO could precipitate under these conditions. First, a 1 M solution in water was created with one of six acids (listed in **Figure** **3**), and 9.1 mmol Sr(OH)_2_.8H_2_O was added into this solution and allowed to dissolve. Next, one of four titanium alkoxide precursors was added into the acidic strontium‐containing solution to create a bimetallic Sr/Ti solution in just water. Finally, this bimetallic solution was added to a 10 M NaOH solution to create the Sr–Ti–OH mixture. A cartoon schematic of this is presented in Figure 3 to aid visualization, and the final compositions of each Sr–Ti–OH mixture as analyzed by powder X‐ray diffraction are presented in Figure 3 as well.

No Sr–Ti–OH mixture created with a moderately strong acid precipitated SrTiO_3_ prior to heating, and final mixture composition was independent of acid p*K*_a_. All Sr–Ti–OH mixtures were amorphous or insoluble byproducts, such as strontium oxalate, or Sr(OH)_2_.8H_2_O (Figure S3c, Supporting Information). These investigations into the phase composition of various Sr–Ti–OH mixtures may clarify why many hydrothermal syntheses for STO nanocuboids utilize TiCl_4_ as a Ti^4+^ source. Most reported hydrothermal syntheses of STO are conducted in unstirred autoclave reactors with convection heating, and under these conditions, it is critical that STO precipitate prior to heating because heat treatment alone is not sufficient to drive STO formation and {100} facet growth.

### 2.2 STO Synthesis with TiCl_4_ Using Microwave‐Assisted Heating

STO support samples were synthesized in a 1 L microwave reactor at 200 °C for 8 h using TiCl_4_ as a Ti^4+^ source to afford products with masses of 12.1 and 21.5 g that contained 80.2% and 82.9% nanocuboids, respectively, with product yields of 90.3% and 80.2%. The output was increased from the 10 g scale to the 20 g scale by doubling the concentration of reagents used in the Sr–Ti–OH mixture. These reaction mixtures were analyzed both prior to and after hydrothermal treatment (Figure S4, S5, Supporting Information) by transmission electron microscopy and powder X‐ray diffraction.

The percentage of nanocuboid particles per sample is around 80% for both syntheses, which is equivalent to the percentages reported for STO samples made by convection heating. The average particle sizes are dependent on starting reagent concentration, and the particle sizes reported in Figure S5, Supporting Information, are consistent with starting reagent concentrations reported for equivalent samples synthesized by convection heating in a 125 mL reactor in Peczak et al. Figure S4, Supporting Information, shows powder X‐ray diffraction patterns and electron microscopy images of the prehydrothermal Sr–Ti–OH mixtures used for STO synthesis at the 1 L scale. These gel mixtures were analyzed and compared to Sr–Ti–OH mixtures synthesized at the 125 mL scale. Precipitation of SrTiO_3_ nanocrystals is still observed, and the average size of these crystallites is equivalent to that observed at the 125 mL scale. The morphology of the precipitated STO particles is irregular as observed in earlier works.

**Figure 4:**
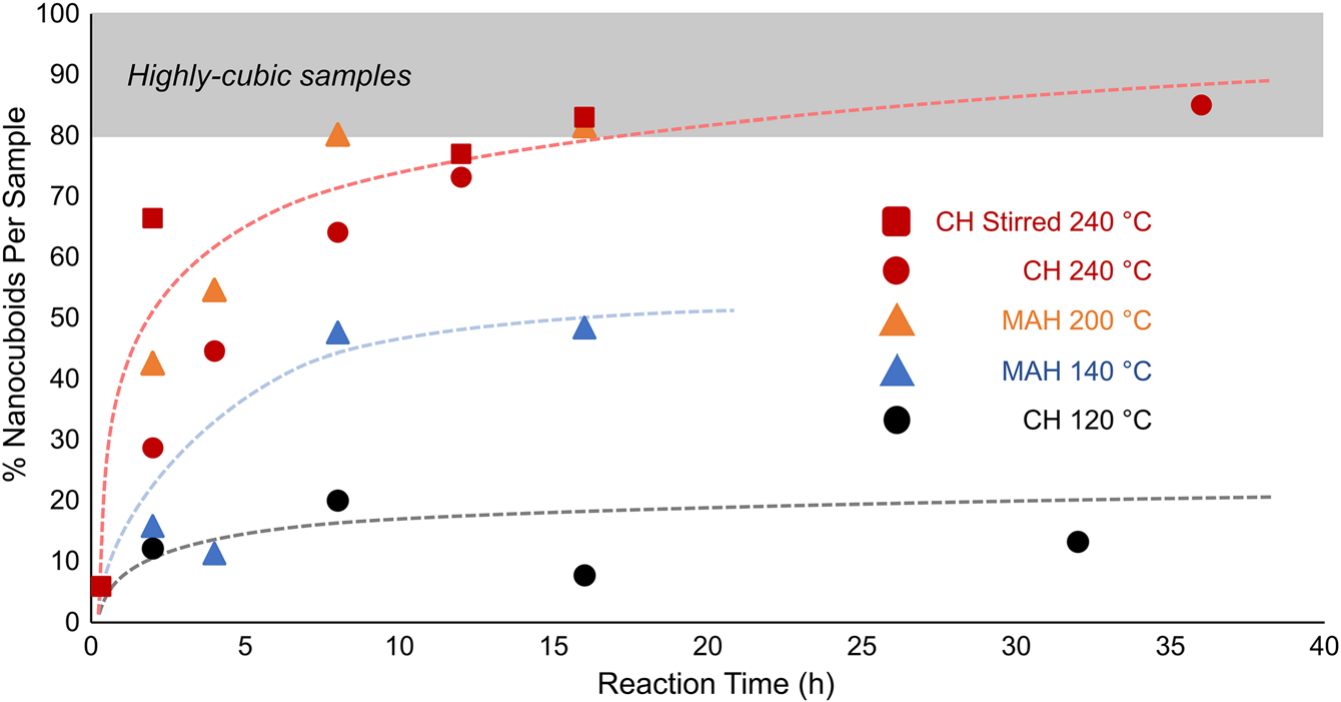
Percent nanocuboid particles for STO samples synthesized by either microwave heating (MAH–STO) or convection heating (CH–STO) with and without stirring at various temperatures and times. The percent nanocuboids per sample appear to evolve similarly independent of heating method. Red circular markers are used for CH–STO data at 240 °C, triangular markers are used for MAH–STO data, and black circles are used for CH–STO data collected at 120 °C. For each data point, 300 nanoparticles were analyzed to determine the percent nanocuboids per sample. The dashed lines are included to guide the reader.

As previously stated, because a beveled nanocuboid is the Wulff shape of STO in water, under sufficiently high temperatures and long reaction times, all SrTiO_3_ nanoparticles in a water‐based reaction medium should rearrange to this shape.^[^ ^82^, ^83^, ^84^ ^]^ To confirm this, irregularly shaped STO nanoparticles were introduced into a pH 14 water–ethanol solution and heated hydrothermally (240 °C, 72 h). After washing, filtering, and drying, the final product was observed to be highly cubic (Figure S6, Supporting Information). However, to further optimize the STO synthesis in pursuit of commercial viability, it is important to understand how the amount of nanocuboid particles that form per sample is affected by reaction time, reaction temperature, heating method, and stirring. Specifically, it is important to identify the minimum temperature and time required for synthesizing highly cubic samples using both microwave heating (MAH) and convection heating (CH). Synthesizing STO materials at this minimum temperature (i.e., optimizing the temperature) will facilitate future development of an economically viable STO synthetic process. Thus, STO samples were synthesized over a range of reaction times and at several temperatures using stirred microwave heating, unstirred convection heating, and stirred convection heating in a 4 L batch reactor. In each case, average particle sizes and percent nanocuboids per sample were measured. Average STO particle sizes are tabulated in Table S2 and S3, Supporting Information, and a graph of percent nanocuboids per sample versus time for each condition is presented in **Figure** **4**. A version of Figure 4 containing information on average particle size is presented in Figure S7, Supporting Information. Moreover, to aid visualization of the results conducted in this work, the experimental design used in this work is presented in Table S1a,b, Supporting Information. Here, the three heating profiles tested on all Sr–Ti–OH reaction mixtures are presented in Table S1a, Supporting Information, as are lists of all seven Ti^4+^ sources and seven Sr^2+^ sources used to synthesize Sr–Ti–OH mixtures (Table S1b, Supporting Information).

The final average STO particle size appears slightly smaller for samples synthesized with microwave heating and stirred convection heating than for unstirred convection heating (between 50 and 60 nm for MAH samples and 42 and 59 nm for stirred CH samples, versus between 55 and 75 nm unstirred CH samples). The variance in average size appears generally equivalent across all samples. The smaller average size of the prehydrothermal STO particle (Figure S4, Supporting Information) may explain why MAH–STO samples are smaller than CH–STO samples because based on previous reports, facet growth appears to increase particle size by around 15–30% of the preheating particle size.^[^ ^24^ ^]^ Beyond observations of differences in average particle size, we have previously established that varying the concentration of Sr^2+^, Ti^4+^, and OH^−^ in solution affords control over final nanocuboid average size, which determines the surface area per gram support available for deposition. In general, increasing Sr^2+^, Ti^4+^, and OH^−^ concentrations drives formation of smaller nanocuboids. By applying this methodology to the microwave‐assisted heating methods developed herein, MAH–STO could be synthesized both on 10 and 20 g scales with average sizes between 20 and 80 nm.^[^ ^24^ ^]^

To synthesize a highly cubic STO sample, a prehydrothermal mixture must be treated for 8 h at 200 °C when using microwave‐assisted heating, compared to around 18 h at 240 °C when using unstirred convection heating. In a 4 L, stirred convection batch reactor at 240 °C, the Sr–Ti–OH reaction mixture must be treated for around 12 h to attain said highly cubic sample. This is less time than is required for an unstirred reactor, but these reaction conditions are still harsher than those used for microwave heating (8 h at 200 °C). Thus, while both stirring and heating increase energy distribution throughout a reaction system, microwave heating seems to have a larger contribution toward speeding up nanocuboid formation than does stirring. Moreover, increased energy distribution in microwave heating likely facilitates STO crystallite precipitation and {100} facet growth at lower temperatures than is possible in convection heating.

For unstirred samples at lower temperatures (120 °C for CH, 140 °C for MAH), reaction times of up to 36 h for unstirred convection heating and 16 h for microwave heating were insufficient for the formation of highly cubic samples. Moreover, unstirred convection heating of a Sr–Ti–OH mixture at 120 °C for 64 h was also insufficient to form a highly cubic sample, producing only 10.6% nanocuboids (Figure S8, Supporting Information). At higher temperatures (240 °C for CH, 140 °C for MH), the evolution of percent nanocuboids per sample versus reaction time is equivalent for both heating methods. Large increases in the percent nanocuboids per sample are observed early on, and eventually, additional time causes less pronounced changes in the percent nanocuboids per sample, which stops increasing at around 80–90% depending on the batch. The differences in sizes between average SrTiO_3_ crystallites and final nanocuboid size are comparable to those observed in previous work.^[^ ^24^ ^]^ This suggests that in both convection heating and microwave‐assisted heating syntheses, STO nanocrystals precipitate prior to heat treatment, and {100} facets grow directly on individual particles during hydrothermal heating, with limited diffusion between particles. As previously noted by Peczak et al., the size of these precipitated STO nanocrystals heavily influences final STO nanocuboid size.^[^ ^24^ ^]^ Facet growth appears to increase particle size by up to 30% from that of the precipitated crystallite, but not further. As a result, changing heating method, reaction time and temperature, may change the progression toward a final nanoparticle size, but not change its value. This is not the case with other materials, where higher temperatures and longer reaction durations can influence increase particle size.

Finally, elemental analysis of STO synthesized on 1, 10, and 20 g scales shows that all reported samples contain Sr and Ti in around a 1:1 ratio independent of STO output (Table S4, Supporting Information). This confirms that increasing reaction scale likely does not create any dispersion‐related issues that could impact STO precipitation and facet growth. Moreover, deficiencies of Sr and Ti in SrTiO_3_ often lead to the formation of extended structures and Ruddlesden–Popper phases, such as Sr_2_TiO_4_. Examples of this are well documented for SrTiO_3_.^[^ ^84^, ^85^ ^]^ These results all suggest that the chemistry and mechanisms governing the formation of STO nanocuboids are independent of the heat treatment method used. Thus, MAH STO samples will likely have surface chemistry comparable to that of previously synthesized CH STO samples, making them viable candidates for Pt/SrTiO_3_ catalyst synthesis.

### 2.3 Microwave STO Synthesis without Ethanol with TiBALD as a Ti^4+^ Source

**Figure 5:**
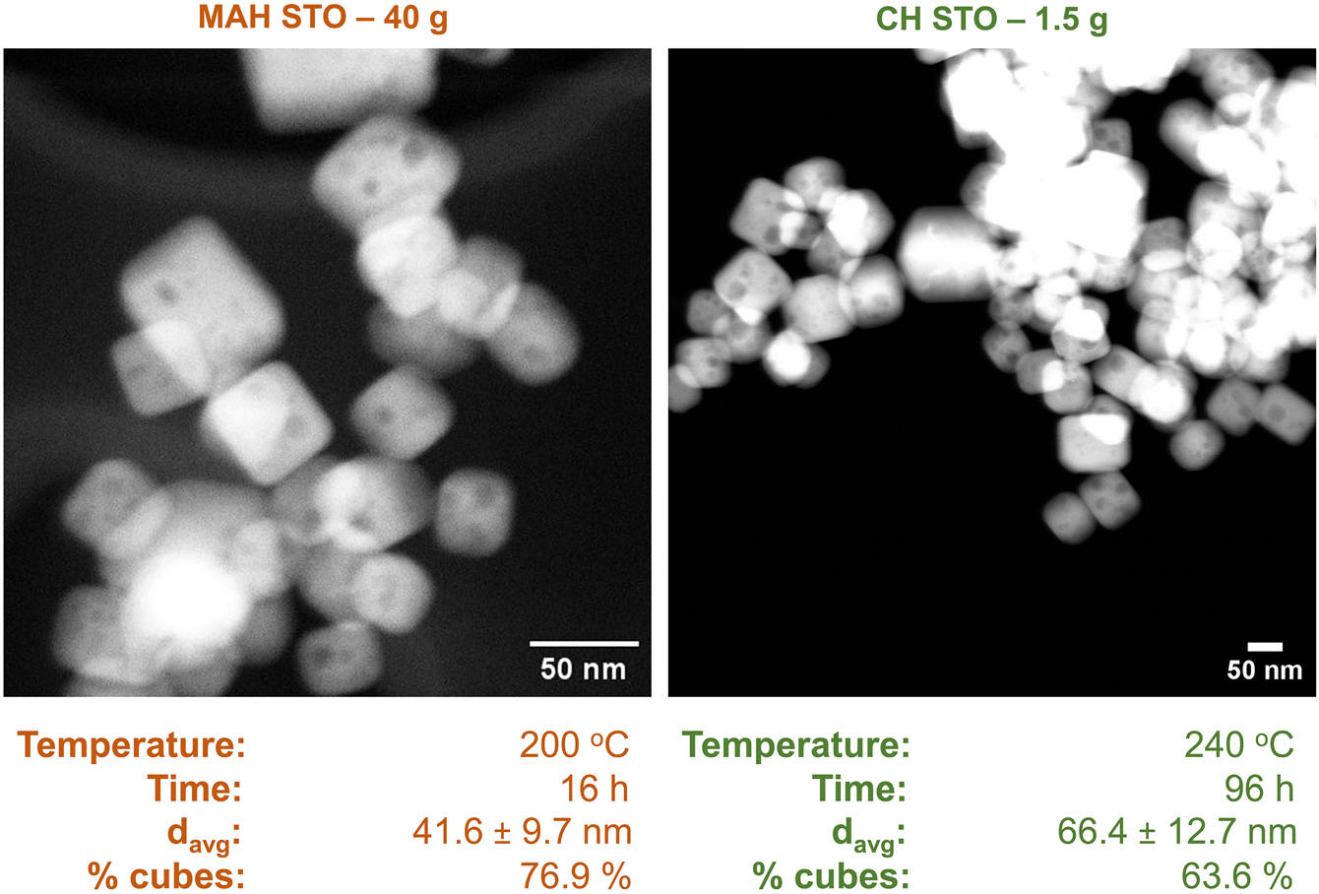
HAADF STEM images of STO supports synthesized by microwave‐assisted heating and convection heating using TiBALD as a Ti^4+^ source. Both samples are >99.9% STO by powder X‐ray diffraction. While the STO sample treated by MAH was highly cubic after heating, the unstirred CH sample was not highly cubic despite being treated at higher temperatures and longer reaction times. For both the MAH–STO and CH–STO samples, 200 nanoparticles were analyzed to determine average nanocuboid size, and 300 nanoparticles were analyzed to determine the percent of nanocuboids in the sample.

Next, a new synthetic route to STO supports was created by replacing TiCl_4_ and ethanol with an equimolar amount of [NH_4_CH_3_CH(O–)CO_2_]_2_Ti(OH)_2_] (TiBALD). This new Sr–Ti–OH mixture was treated in both a microwave reactor and a convection hydrothermal oven, and the resulting samples were analyzed by both electron microscopy and X‐ray diffraction (**Figure** **5** and Figure S7, Supporting Information).

In both reactions, the final product is >99.9% crystalline SrTiO_3_ with no secondary phases (Figure S9, Supporting Information). When using convection heating, treatment at 240 °C for 96 h was insufficient to produce highly cubic STO, with the final percent of nanocuboids in the sample reaching 63.6%. By contrast, microwave heating at 200 °C for 16 h afforded a final STO sample with 76.9% nanocuboids, which is slightly less than required for a highly cubic STO sample (80% nanocuboids). This is likely one of the first reports of an STO hydrothermal synthesis that produces a cubic final sample on a 20 + g scale without the use of TiCl_4_ as a Ti^4+^ source. The pre‐Sr–Ti–OH reaction mixture for this sample was amorphous despite having a final pH of 14 (Figure S10, Supporting Information). It is likely that for this reaction system, microwave heating and stirring are sufficient to allow both the precipitation of STO and facet growth during heating. This is not the case when the sample is treated via convection heating, which is consistent with previous observations about the impact of Sr–Ti–OH mixture pH variation and STO precipitation on final particle morphology.

## 3 Conclusion

The synthesis of SrTiO_3_ nanocuboid supports was investigated to both understand the composition of the Sr–Ti–OH reaction mixture prior to heating and determine the impact of heating on STO particle morphology by developing a synthetic route based on microwave heating. Phase stability studies of various Sr–Ti–OH mixtures prior to heating demonstrate STO nanocuboid formation proceeds through a two‐step precipitation and facet growth process. When any Ti^4+^ source other than TiCl_4_ is used, STO does not precipitate prior to heating because titania sol–gel structures are more thermodynamically stable. This is likely because the solution pH increased to an intermediate range (3 < pH < 9). When one‐step convection heating profiles are used, STO must precipitate prior to heating to obtain a highly cubic sample. Next, the impact of heating method chosen on the final STO sample was investigated by testing microwave‐assisted heating for hydrothermal treatment. Two syntheses were developed that produce around 20 g highly cubic STO under milder conditions than previously possible (200 °C, up to 16 h), using both TiCl_4_ and titanium(IV) bis(ammonium lactato)dihydroxide as Ti^4+^ reagents. The latter is one of the first examples of a synthesis for highly cubic STO without the use of titanium tetrachloride and ethanol. Overall, the results demonstrate that rational design of STO catalyst supports for commercial applications is possible. The insights gained from investigating how different Ti^4+^ sources impact STO formation may be also applied to other materials such as lithium titanium oxide anodes, whose syntheses also rely on similar reagents.^[^ ^86^, ^87^, ^88^ ^]^

## 4 Experimental Section

4.1

4.1.1

##### Materials and Instrumentation

Samples were analyzed by powder X‐ray diffraction and electron microscopy as described previously in Peczak et al.^[^ ^24^ ^]^

##### Inductively Coupled Plasma Optical Emission Spectrometry

Elemental Sr and Ti compositions in STO were measured by a Thermo iCAP 7600 inductively coupled plasma optical emission spectroscopy (ICP‐OES) system operated through the Quantitative Bio‐element Imaging Center at Northwestern University. STO samples (40 mg) were digested in aqua regia (15 mL) for 48 h, after which 0.5 mL of the resulting solution was diluted 20‐fold with a 2% HNO_3_/HCl solution in water (10 mL total sample volume) and analyzed. Five stock solutions were made by serial dilution from Sr and Ti standards to span a range of 1–40 ppm for each metal for calibration. A calibration curve was computed internally through the ICP‐OES system software. Final concentration data were converted to molar amounts and divided to obtain a value of the Sr/Ti ratio. Standard deviation of the Sr/Ti ratio was computed through error propagation of the standard deviation associated with the measurements of Sr and Ti concentrations in the ICP sample. The details of this calculation are presented below in the Statistical Analysis subsection.

##### SrTiO_3_ Synthesis: Safety Note

Handle with care. Perchlorate salts are a class of potentially explosive chemicals when subjected to certain conditions. These chemicals can release a destructive amount of pressure, gas, or heat when subjected to certain conditions such as high temperature or source of ignition. These compounds also tend to be strong oxidizers. Contact with other materials may cause and/or intensify fires.

All SrTiO_3_ nanoparticle samples were obtained by first synthesizing a Sr–Ti–OH mixture and then heating it (Equation (1)). Sr–Ti–OH mixtures were synthesized from various Sr^2+^ sources (SrCO_3_, Sr(C_2_H_4_O_2_)_2_, Sr(NO_3_)_2_, Sr(OH)_2_.8H_2_O, Sr(C_2_H_4_O_2_)_2_.0.5H_2_O, Sr(ClO_4_)_2_.3H_2_O, SrCl_2_.6H_2_O), and various Ti^4+^ sources (TiCl_4_, Ti(OCH_2_CH_3_)_4_, Ti(OC_3_H_7_)_4_, Ti(OCH(CH_3_)_2_)_4_, Ti(OC_4_H_9_)_4_, Ti(OC(CH_3_)_3_)_4_, [NH_4_CH_3_CH(O‐)CO_2_]_2_Ti(OH)_2_]). Hydrothermal heating was done either by convection heating or microwave heating (Equation (1)). Both techniques were used to treat Sr–Ti–OH mixtures synthesized on various scales.

(1) $$\text{(Sr}–\text{Ti}–{\text{OH mixture synthesis) + (heating method) =SrTiO}}_{3}\text{nanoparticles}$$

##### Sr–Ti–OH Mixture Creation with Ethanol: Lab Scale

Solution A: As‐received Sr^2+^ reagents (10.0 mmol, Sigma–Aldrich) were added to acetic acid (0.05 mol, Sigma–Aldrich, 99.5%) in deionized water (50 mL) and stirred for 10 min.

Solution B: As‐received Ti^4+^ reagents (9.1 mmol, Sigma–Aldrich) were added to ethanol (20 mL, absolute) in a 50 mL beaker via Luer Lock syringe and stirred for 10 min. Solution A and Solution B were combined to make Solution AB and stirred for 10 min to ensure homogeneity. Next, Solution AB was added into a NaOH solution (13 mL, 10 M) to synthesize the designated Sr–Ti–OH mixture. In situations where a final nanoparticle synthesis was pursued, this Sr–Ti–OH mixture was transferred in its entirety to a hydrothermal reactor and heated, as described below. In the case of gel precursor analyses without hydrothermal heating, this Sr–Ti–OH mixture was transferred to a 50 mL Falcon tube. It was then centrifuged at 4500 rpm for 5 min, after which the white gel at the bottom of the centrifuge tube was separated from the supernatant, spread thin on a crucible, and analyzed by powder X‐ray diffraction or electron microscopy. This was done immediately after separation to avoid gel decomposition in air. This method is derived from the process published by Peczak et al.^[^ ^24^ ^]^

##### Sr–Ti–OH Mixture Creation with Ethanol: Large Scale

Sr‐Ti‐OH mixtures for large‐scale, convection‐based hydrothermal synthesis in a 4 L reactor were conducted as described previously in Peczak et al.^[^ ^24^ ^]^

##### Sr–Ti–OH Mixture Creation without Ethanol: Lab Scale

As‐received Sr^2+^ reagents (9.1 mmol, Sigma–Aldrich) were added into a 1 M solution of acetic acid (0.05 mol, Sigma–Aldrich, 99.5%) in deionized water (50 mL) and stirred for 10 min to ensure dissolution. Next, an as‐received Ti^4+^ source (9.1 mmol, Sigma–Aldrich) was added to the Sr^2+^ solution, and the resulting Sr/Ti bimetallic solution was allowed to stir for 10 min. Depending on which Ti^4+^ source was used, either a white precipitate formed, a polymeric network formed, or no solid formed. The Sr/Ti bimetallic solution was next added to a NaOH solution (10 M, 30 mL) and stirred for 10 min to form the final Sr–Ti–OH mixture. In situations where a final nanoparticle synthesis was pursued, this Sr–Ti–OH mixture was transferred in its entirety to a hydrothermal reactor and heated, as described below. In the case of gel precursor analyses without hydrothermal heating, this Sr–Ti–OH mixture was transferred to a 50 mL Falcon tube and isolated for analysis as described above.

##### Sr–Ti–OH Mixture Creation without Ethanol: Large Scale

Only one Sr–Ti–OH mixture was synthesized without ethanol on a scale larger than 125 mL. Sr(OH)_2_.8H_2_O (77.8 g, 0.29 mol) was added into a solution of acetic acid (71.7 g, 1.20 mol) and double deionized water (450 g) in a 1 L beaker and stirred for 10 min. [NH_4_CH_3_CH(O‐)CO_2_]_2_Ti(OH)_2_ (50% by weight solution in water, Sigma–Aldrich, 172.18 g, 0.59 mol) was added into this solution, which then turned yellow. The resulting Sr/Ti bimetallic solution was allowed to stir for 10 min, after which it was added into a separate 1 L beaker containing 10 m NaOH (316 mL, 3.16 mol). The resulting gel was stirred for 15 min.

##### Hydrothermal Synthesis by Convection Heating

Convection‐based hydrothermal heating at both the 125 mL scale and 4 L scale were conducted as described in Peczak et al.^[^ ^24^ ^]^

##### Hydrothermal Synthesis by Microwave Heating

Microwave experiments were conducted in a Milestone synthWAVE Single Reaction Chamber reactor. A predetermined Sr–Ti–OH mixture (≈800 mL) was transferred to a 1 L Teflon liner, which was inserted into the synthWAVE reactor. A heat treatment was applied consisting of 1) a 30 min ramp to a selected temperature (120–240 °C); 2) a hold time of 2–16 h with 50% maximum stir speed; and 3) a 30 min cool step back to ambient temperature. The resulting white precipitate was washed repeatedly with deionized water via vacuum filtration until the water in the Buchner funnel was pH 7. The wet powder was then removed from the funnel and dried in air in an oven (110 °C, overnight).

##### Statistical Analysis

All data presented in this work were processed as collected without any preprocessing, normalization, or removal of outliers. Data corresponding to average particle sizes are presented as the mean and standard deviation of values measured. For each measurement of average size, around 200 nanoparticles were counted. Percentages of nanocuboids per sample were calculated by counting the number of particles identified as “nanocuboids,” and dividing by the total number of particles observed in the corresponding microscopy image (see below). For each calculation of the percent nanocuboids in a sample, 300 nanoparticles were considered and identified as either “nanocuboids” or “not nanocuboids” (Equation (2)). Calculations of average particle size and percent nanocuboids were conducted in Microsoft Excel, and the relevant data were collected using ImageJ.

(2) % Nanocuboids = (# Nanocuboids Observed) / (# Total Particles Observed)

Average crystallite size for powder X‐ray diffraction samples was calculated using the Debye Scherrer equation within the MDI Jade software package.^[^ ^87^ ^]^ Standard deviation associated with Sr/Ti ratio calculations (***r***) was measured by error propagation of the standard deviation associated with the Sr and Ti concentrations measured by ICP‐OES as demonstrated below (Equation (3)–(5)). Equation (3) and (4) were used to calculate the standard deviation associated with mass and molar amounts of both Sr and Ti, but are shown below only for Sr for conciseness.

(3) $$\sigma_{\text{mass Sr}}=\sigma_{\text{mass Sr}}*10^{-6}*0.04g$$

(4) $$\sigma_{\text{mol Sr}}=\frac{\sigma_{\text{mass Sr}}}{\text{Molar Mass Sr}}$$

(5) $$\sigma_{r}=r\sqrt{{\left(\frac{\sigma_{\text{mol Sr}}}{\text{mol Sr}}\right)}^{2}+{\left(\frac{\sigma_{\text{mol Ti}}}{\text{mol Ti}}\right)}^{2}}$$

## Conflict of Interest

The authors declare no conflict of interest.

## Author Contributions

The manuscript was written through the contributions of all authors. All authors have given approval for the final version of the manuscript.

## Supporting information

Supplementary Material

## Data Availability

The data that support the findings of this study are available from the corresponding author upon reasonable request.
