# Supplementary material for: Microwave‐Assisted Synthesis of SrTiO3 Nanocuboids without TiCl4
Source: Small Sci. 2023 Apr 6;3(5):2200107. doi: 10.1002/smsc.202200107 (PMC11935966; doi:10.1002/smsc.202200107)
Supplement: Supplementary file 1 — Supplementary Material [file SMSC-3-2200107-s001.pdf]

Supplementary Information

Microwave-Assisted Synthesis of $SrTiO_3$ Nanocuboids without $TiCl_4$

Authorship: *Ian L. Peczak, Robert M. Kennedy, Aili M. Simpson, Massimiliano Delferro, Kenneth R. Poeppelmeier*

Corresponding author

Kenneth Poeppelmeier: krp@northwestern.edu

Table of Contents

1. Schematic of Sr-Ti-OH Reaction Mixture Screening Method……………………...S2
2. Diffraction Patterns of Sr-Ti-OH Mixtures for Samples from **Figure 1**…………....S3
3. Representative Diffraction Pattern for Amorphous Sr-Ti-OH Mixture…………....S4
4. Representative Diffraction Pattern for Weakly Crystalline Sr-Ti-OH Mixture…….S4
5. Representative Diffraction Pattern for Crystalline Sr-Ti-OH Mixture………..……S5
6. TEM Micrographs of Sr-Ti-OH Mixtures for 125 mL and 1 L Syntheses…………S6
7. Diffraction Pattern of Post-Reaction $SrTiO_3$ Product from MAH Synthesis……….S6
8. HAADF STEM Micrographs of MAH STO from a 1 L Reactor……………………S7
9. HAADF STEM Micrograph of Re-Treated STO Nanocuboid Supports……….....S8
10. Summary of Experimental Design for MAH & CH STO Syntheses………………..S9
11. Average Particle Sizes for MAH & Unstirred CH STO…………………………...S10
12. Average Particle Sizes and Percent Cubes for Stirred CH STO…………………S10
13. Percent Nanocuboids and Corresponding Average Particle Sizes …………….S11
14. STO Samples Treated by Unstirred Convection Heating, 120 ^oC, 64 h………..S12
15. Sr/Ti Ratios for STO Samples Synthesized at 1 g, 10 g, and 20 g scales……….S13
16. Diffraction Pattern of STO Nanoparticles Synthesized Using TiBALD…………S13
17. Diffraction Pattern of Sr-Ti-OH Mixture for TiBALD Synthesis…………………..S14

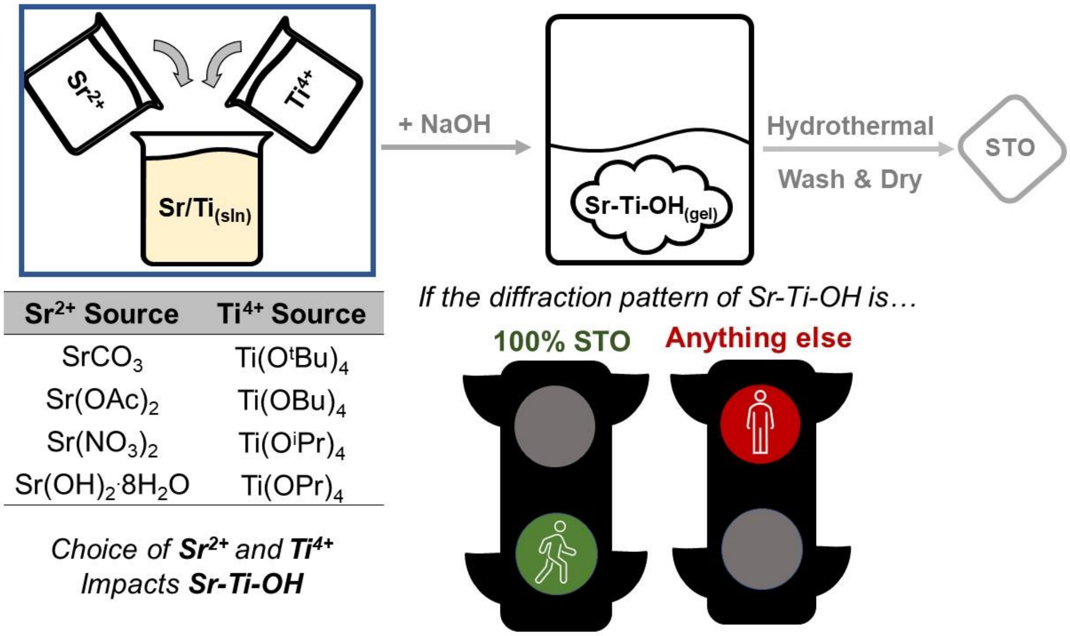

Sr^{2+} Source	Ti^{4+} Source
$SrCO_3$	$Ti(O^tBu)_4$
$Sr(OAc)_2$	$Ti(OBu)_4$
$Sr(NO_3)_2$	$Ti(O^iPr)_4$
$Sr(OH)_2{\cdot}8H_2O$	$Ti(OPr)_4$

Figure S1. Synthesis of Sr-Ti-OH Reaction Mixtures for Screening by X-Ray Diffraction. Sr^{2+} solutions were made in 1 M CH_3COOH with one of the reagents listed in the "Sr^{2+} Source" column, and Ti^{4+} solutions were made in ethanol with one of the reagents listed in the "Ti^{4+} Source" column. These two solutions were combined and introduced into a 10 M NaOH solution. After stirring, the resulting Sr-Ti-OH mixture was centrifuged, and a gel material was removed for analysis by powder x-ray diffraction.

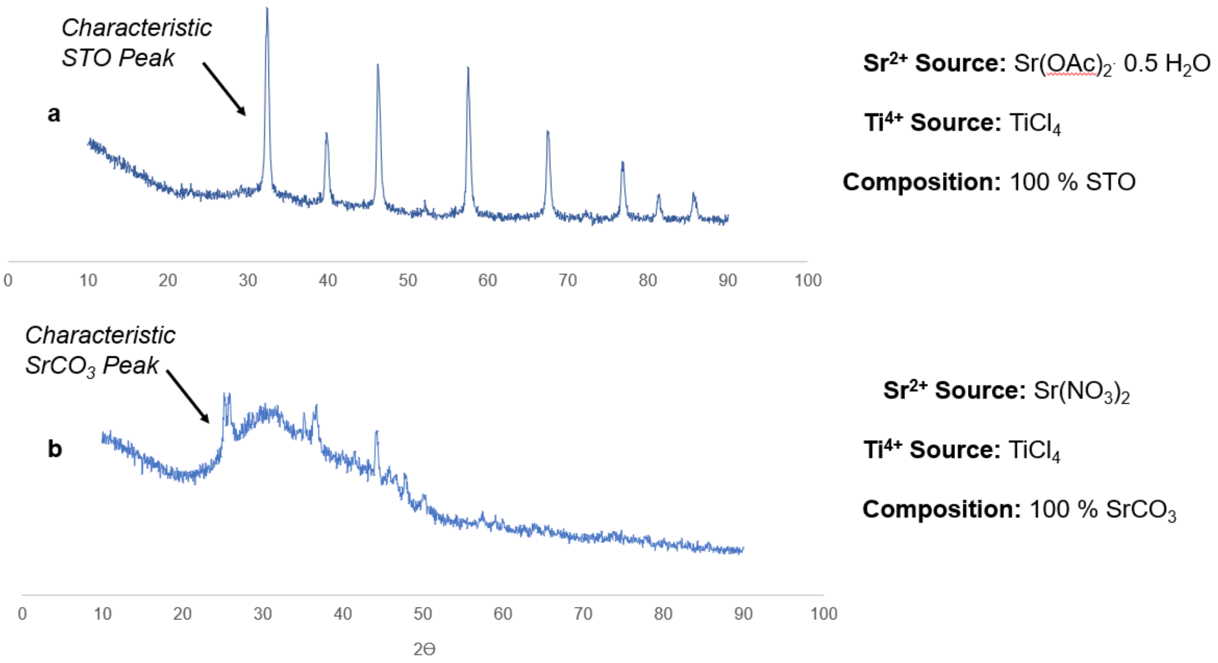

Figure S2. Sr-Ti-OH Mixtures for STO Samples in Figure 1 that were Highly Cubic (a) and Irregular (b) After Heating. The Sr-Ti-OH mixture characterized in **S2a** became a highly cubic STO sample after heating, while the Sr-Ti-OH mixture in **S2b** became an irregular STO sample after heating. The diffraction pattern in **S2a** corresponds to a "100 % STO" pattern that has been shown to make nanocuboids, while the diffraction pattern in **S2b** corresponds to a "weakly crystalline" pattern with a $SrCO_3$ primary phase. The "weakly crystalline" designation was assigned because of the low signal-to-noise ratio shown in the pattern.

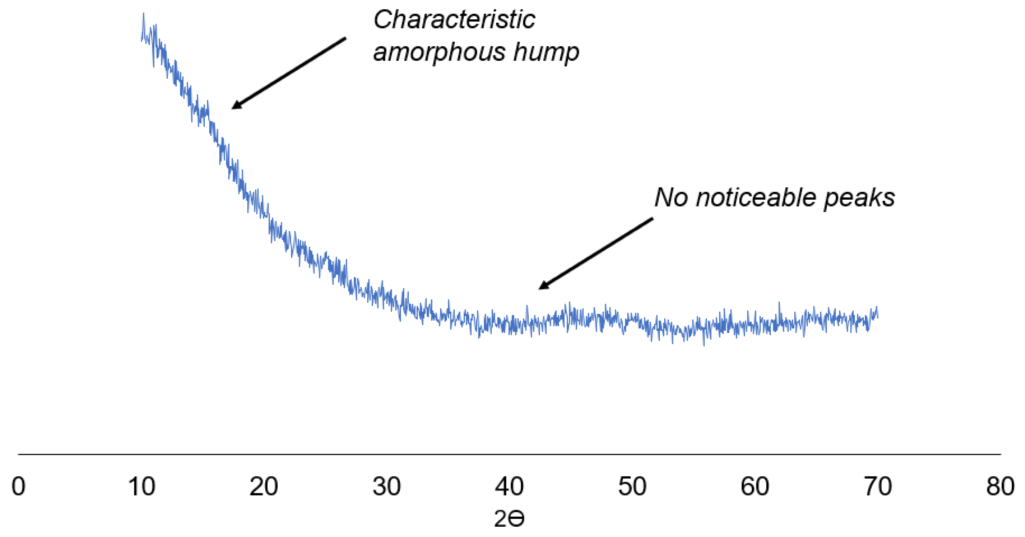

Figure S3a. Representative Powder X-Ray Diffraction Pattern for Sr-Ti-OH samples labeled "Amorphous". In these cases, no crystalline phase is identified in the sample after synthesizing a Sr-Ti-OH gel from the designated Sr^{2+} and Ti^{4+} sources in 10 M NaOH. All Sr-Ti-OH samples designated "amorphous" produced diffraction patterns like this one when characterized.

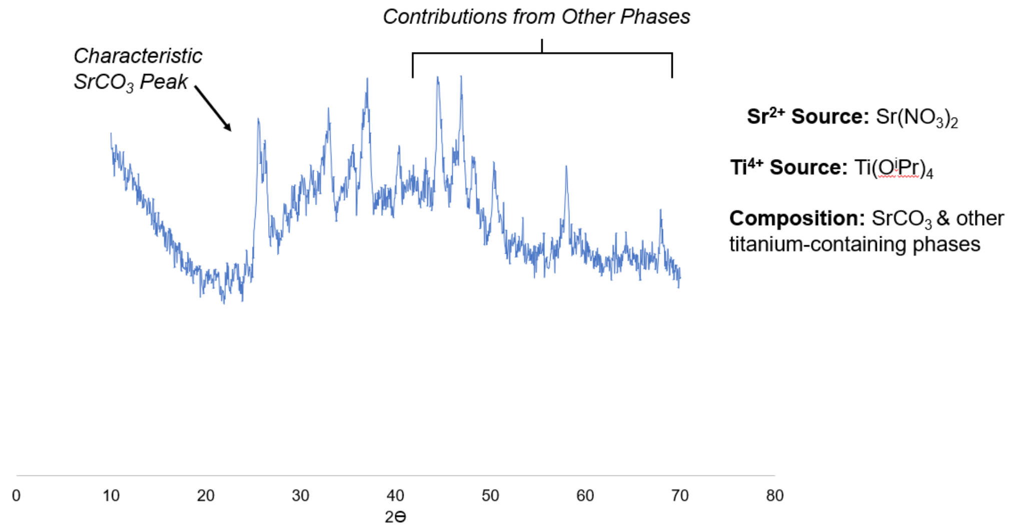

Figure S3b. Representative Powder X-Ray Diffraction Pattern for "Weakly Crystalline" Diffraction Pattern in Figure 2 with Phases other than $SrCO_3$. In such patterns, characteristic peaks corresponding to $SrCO_3$ are observed. However, other peaks are observed in the pattern that are likely due to titanium-containing phases, such as sodium-titanium complexes. The low signal to noise ratio also suggests that a significant portion of the Sr-Ti-OH mixture is amorphous.

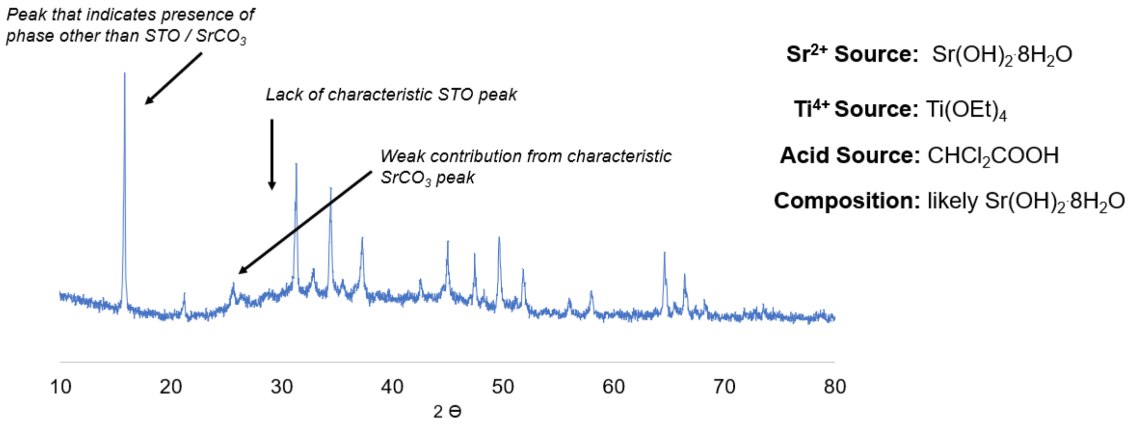

Figure S3c. Representative powder x-ray diffraction pattern for crystalline diffraction pattern where $Sr(OH)_2{\cdot}8H_2O$ is likely the primary phase**.** In these cases, a crystalline phase is observed upon synthesis of the Sr-Ti-OH mixture. However, there is no $SrTiO_3$ in this mixture, and the contribution from $SrCO_3$ does not explain all peaks in the diffraction pattern.

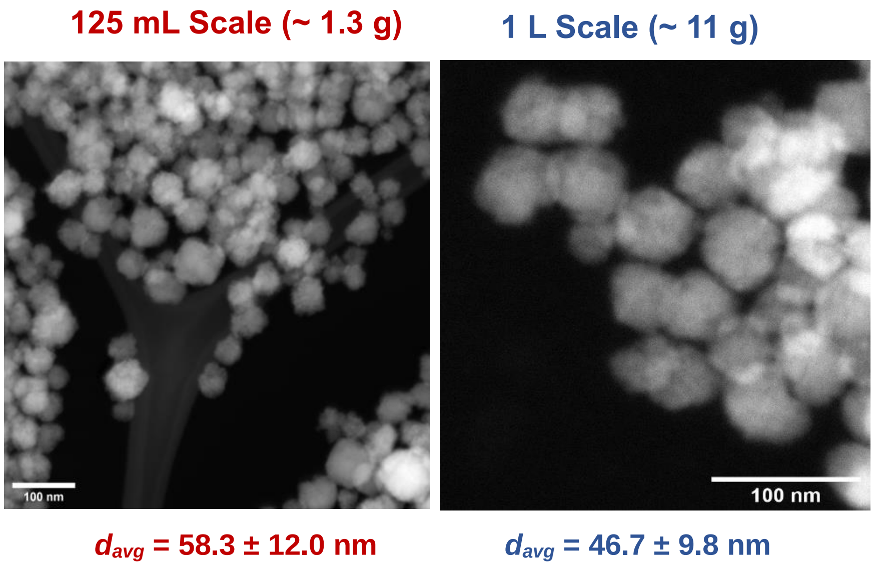

Figure S4a. STEM HAADF Micrographs of Sr-Ti-OH Mixtures Prior to Hydrothermal Treatment For 125 mL and 1 L Scale Syntheses. Both products are 100% crystalline $SrTiO_3$ according to analysis by x-ray diffraction.

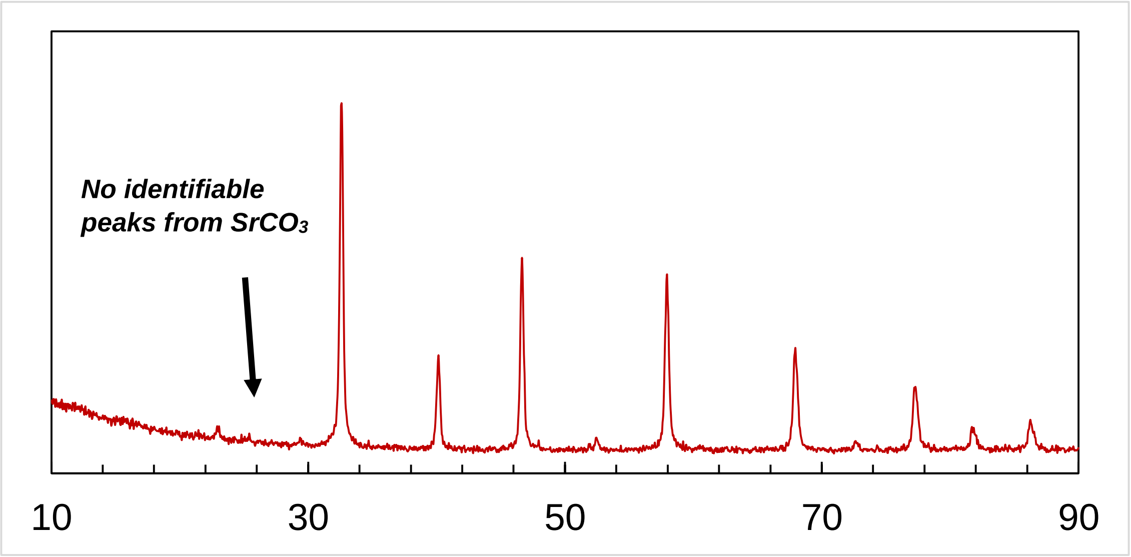

Figure S4b. X-Ray Diffraction Pattern of Post-Reaction MAH STO Product. This powder pattern shows a crystalline sample composed of 100% $SrTiO_3$ and no $SrCO_3$ by-product.

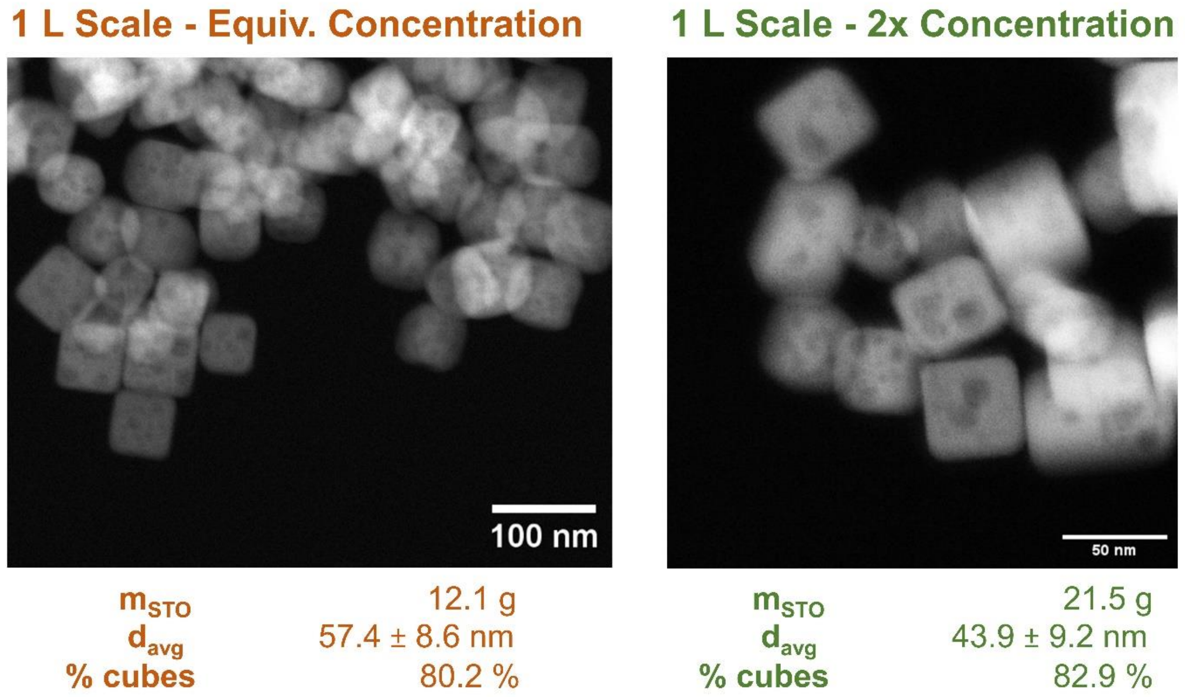

Figure S5. High Angle Annular Dark Field (HAADF) STEM Micrographs of MAH STO Samples Synthesized in a 1L Reactor. Two STO samples were synthesized at equivalent concentrations to the reaction mixture reported by Peczak et al. and at double this concentration, to afford highly cubic STO samples with masses of 12.1 g (90.3 % yield) and 21.5 g (80.2 % yield), respectively. The percentages of nanocuboids per sample appear to be generally equivalent for both the "Equiv. Concentration" and "2x Concentration", and the differences are likely due to batch-to-batch variation. Shapes resembling pores in the STO material are Kirkendall voids internal to each particle and have been observed in previous works. The size decrease across samples is consistent with the previously reported relationship between starting reagent concentration and final particle size.[24]

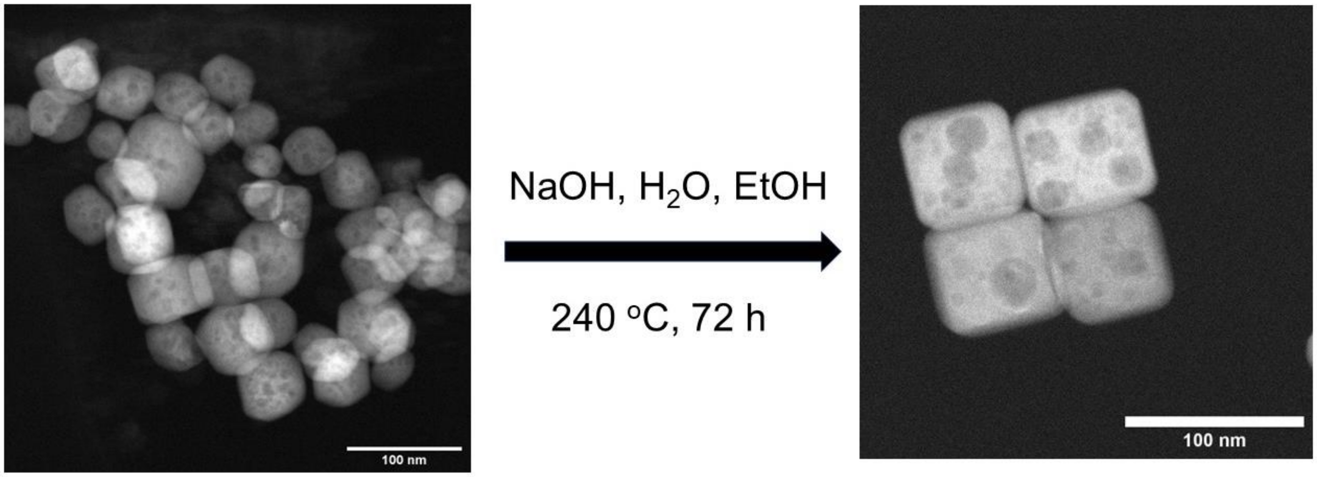

**Mass**	5.0 g
**d_{avg}**	50.4 ± 12.3 nm
**% cubes**	15.2 %

**Mass**	4.8 g
**d_{avg}**	57.2 ± 10.5 nm
**% cubes**	86.5 %

Figure S6. HAADF STEM Micrograph of Re-Treated STO Nanocuboid Supports. 5g of non-cubic STO nanoparticles were introduced into a supernatant with 50 mL water, 20 mL ethanol, and 13 mL of 10 M NaOH, respectively, and treated in a convection-based hydrothermal oven in a 125 mL reactor for 72 h at 240 ^{o}C (1 oC/min ramp, ambient cooling). After centrifuging and washing the sample to pH 7, the final product was analyzed by TEM and shown to be generally equivalent to previous cubic samples.

Table S1. Summary of Experimental Design for STO Synthesis. Presented below are tabulations of the reagents used to make Sr-Ti-OH reaction mixtures (**Equation S1, Table S1a**), and the 3 different heating profiles (**Table S1b, Equation S2**) tested for STO nanoparticle synthesis. Per **Equations S1** and **S2**, all nanoparticles were synthesized by choosing one combination of Sr^{2+} and Ti^{4+} reagents, and one heating method from Tables S1a and S1b, respectively.

Eq. S1: *(Sr^{2+} source) + (Ti^{4+} source) + (NaOH) = Sr-Ti-OH mixture*

***Eq. S2*:** (*Sr-Ti-OH mixture*) + (*heating method*) = *$SrTiO_3$ nanoparticles*

S1a. Sr^{2+} and Ti^{4+} Reagents

Sr^{2+} Sources Used	Ti^{4+} Sources Used
$Sr(OH)_2{\cdot}8H_2O$	$TiCl_4$
$Sr(NO_3)_2$	TiBALD
$Sr(OAc)_2$	$Ti(O^tBu)_4$
$Sr(OAc)_2{\cdot}0.5H_2O$	$Ti(O^tBu)_4$
$Sr(ClO_4)_2{\cdot}3H_2O$	$Ti(OiPr)_4$
$SrCO_3$	$Ti(OPr)_4$
$SrCl_2{\cdot}6H_2O$	$Ti(OEt)_4$

S1b. Heating Methods

Identifier	Heating Method	Max Time (h)	Reactor Volume (L)	Max Temperature (ºC)	Ti^{4+} Source	Output (g)
A	Convection	36	0.125	240	$TiCl_4$	1.5
B	Convection - Heating Jacket	16	4	240	$TiCl_4$	20
C	Microwave Radiation	16	1	200	$TiCl_4$	20
D	Microwave Radiation	16	1	200	TiBALD	20

Table S2. Average Particle Sizes for STO Nanoparticles Synthesized at Varied Times and Temperatures by Both Microwave Heating (*MAH-STO*) and Convection Heating (*CH-STO*).

MAH-STO			CH-STO		
Temperature	Time (h)	d_{avg} (nm)	Temperature	Time (h)	d_{avg} (nm)
140 °C	2	50.4 ± 12.3	120 °C	2	75 ± 20.3
	4	48.2 ± 11.3		8	61.7 ± 12.2
	8	49.6 ± 8.6		16	70.0 ± 14.6
	16	51.3 ± 13.9		32	54.7 ± 11.8
200 °C	2	47.3 ± 7.8	240 °C	2	54.4 ± 12.1
	4	47.1 ± 10.0		4	65.4 ± 13.4
	8	57.4 ± 8.6		8	64.2 ± 14.8
	16	58.9 ± 10.5		12	54.8 ± 9.8
				36	65 ± 10

Table S3. Average Size and % Cubes per Sample for STO Samples Synthesized in a 4 L Hydrothermal Reactor. Average STO particle size varied from 42.3 nm to 58.6 nm, while the average percent of cubes per sample increased from 5.9 % after 20 minutes, to 83 % cubes after 16 hours.

Time (h)	d_{avg} (nm)	% Nanocuboids
0.33	42.3 ± 9.1	5.9
2	49.6 ± 9.9	66.4
12	58.6 ± 16.2	77
16	53.4 ± 12.9	83

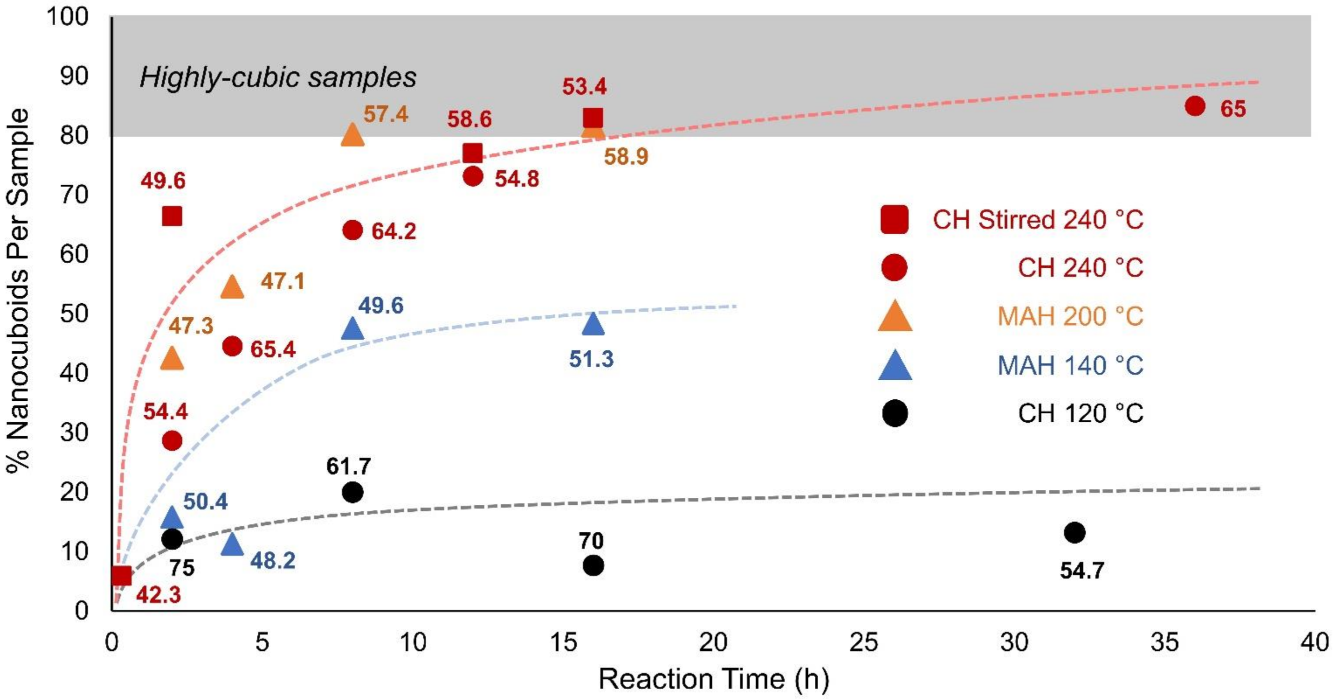

Figure S7. Percent Nanocuboid Particles and Average Particle Sizes for STO Samples Synthesized by Either Microwave Heating (*MAH-STO*) or Convection Heating (*CH-STO*) with and without Stirring at Various Temperatures and Times. This figure is equivalent to **Figure 4** in the main text. However, data labels (number closest to each point) have been added to show the average particle size (in nanometers) of each STO sample analyzed. At least 200 nanoparticles were measured to compute a mean value for each data point.

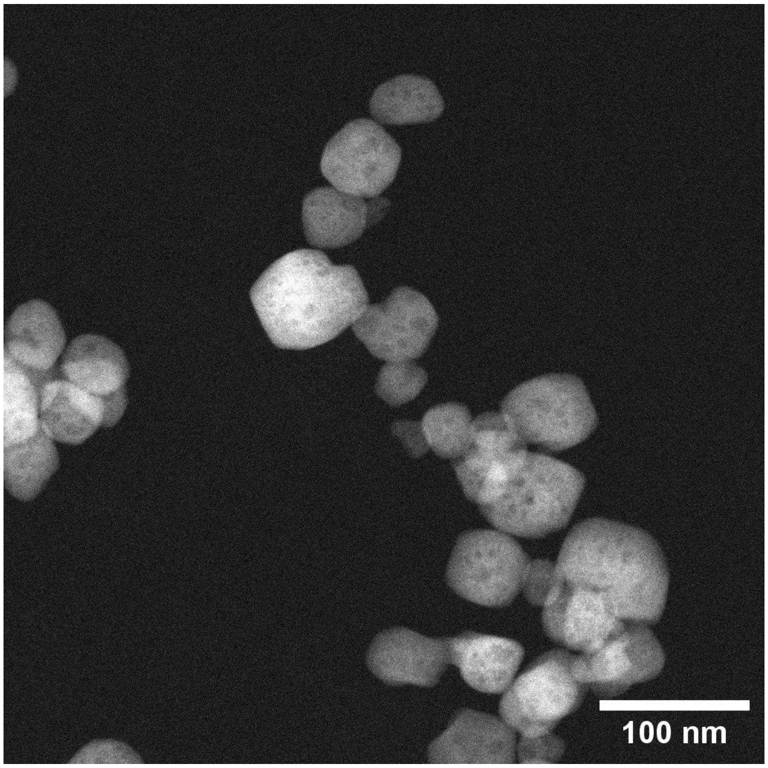

**d_{avg}**	42.7 +/- 10.8 nm
**% Cubes**	10.6 %

Figure S8. Electron Micrograph of $SrTiO_3$ Samples Treated Hydrothermally for 64 hours at 120 ^oC Under Previously Reported Lab Scale Conditions with Convection Heating. These samples do not meet the threshold for "highly cubic" established by Peczak et al as they have 10.6% cubes per sample. The average particle size is 42.7 ± 10.8 nm.

Table S4. Elemental ratio of Sr/Ti for STO samples synthesized on 1 g, 10 g, and 20 g scales. Sr/Ti ratios were calculated based on Sr and Ti concentrations obtained from measurements by inductively coupled plasma optical emission spectrometry (ICP-OES). Standard deviations were calculated by error propagation techniques. The details of these calculations are presented in the *Statistical Analysis* subsection of the Experimental section.

Sample	Scale	Sr:Ti
CH-STO	1 g	1.04 ± 0.06
MAH-STO	10 g	0.96 ± 0.02
MAH-STO-TiBALD	20 g	0.98 ± 0.01

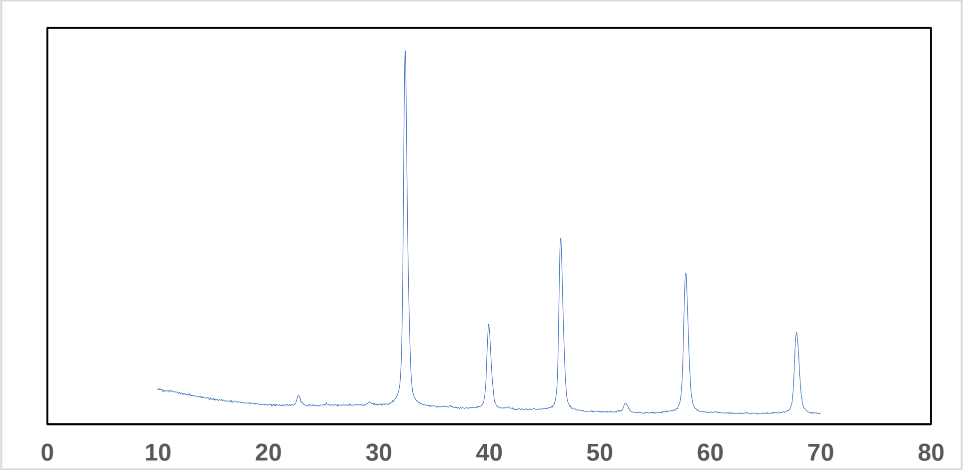

Figure S9. Powder X-Ray Diffraction Pattern of STO Nanoparticles Synthesized Using TiBALD. The final STO sample is 100% crystalline STO with no secondary phases detectable by PXRD.

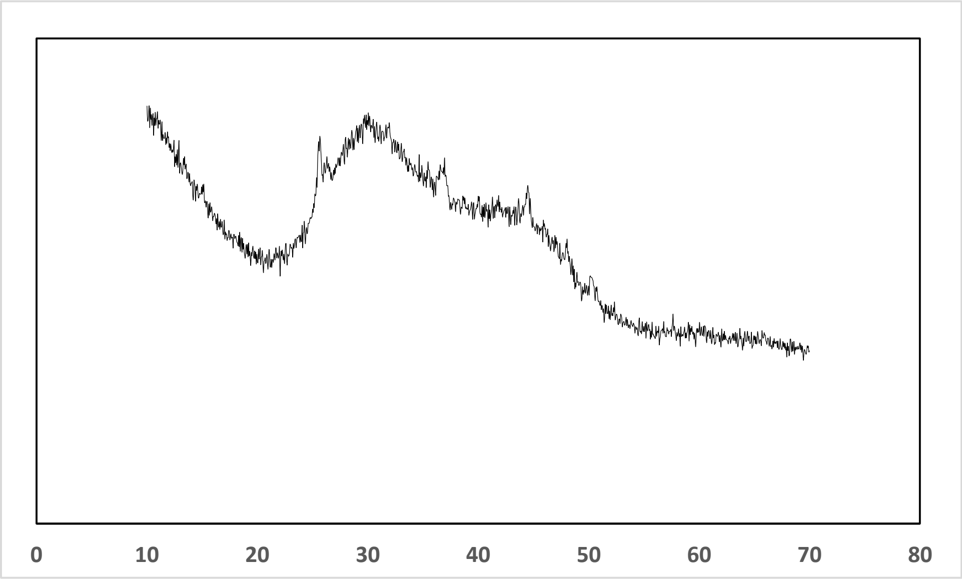

Figure S10. Powder X-Ray Diffraction Pattern of Sr-Ti-OH Gel Precursor Synthesized with TiBALD as a Ti^{4+} Source. The precursor sample is largely amorphous with traces of weakly crystalline $SrCO_3$.
